# Complete Genome Sequence of Vibrio mediterranei 117-T6, a Potentially Pathogenic Bacterium Isolated from the Conchocelis of Pyropia spp.

**DOI:** 10.1128/MRA.01569-18

**Published:** 2019-01-17

**Authors:** Qiqin Liu, Mengya Xu, Yingyun He, Zhen Tao, Rui Yang, Haimin Chen

**Affiliations:** a Key Laboratory of Marine Biotechnology of Zhejiang Province, Ningbo University, Ningbo, Zhejiang, China; b School of Fisheries, Zhejiang Ocean University, Zhoushan, Zhejiang, China; Georgia Institute of Technology

## Abstract

Vibrio mediterranei is a Gram-negative bacterium of the family Vibrionaceae. Vibrio mediterranei strain 117-T6 was pathogenic to Pyropia yezoensis, a red seaweed cultivated in China, by causing death to its conchocelis. Here, we report the complete genome sequence of Vibrio mediterranei 117-T6.

## Abstract (precis)

Vibrio mediterranei is a Gram-negative bacterium of the family Vibrionaceae. Vibrio mediterranei strain 117-T6 was pathogenic to Pyropia yezoensis, a red seaweed cultivated in China, by causing death to its conchocelis.

## ANNOUNCEMENT

The genus Vibrio is a major group of the family Vibrionaceae, and its members are widespread in marine environments, or associate with hosts in those environments, and can be pathogenic or nonpathogenic (1, 2). Since Vibrio mediterranei was first described by Pujalte and Garay in 1986 (3), the bacterium has been isolated from various marine organisms, such as plants, shellfish, other marine invertebrates, and fish from different geographic regions (4 – 6). V. mediterranei 117-T6 was isolated from the bleached shell-born conchocelis of Pyropia yezoensis which was suffering from the fatal disease known as yellow spot disease. The strain was then sent to the China General Microbiological Culture Collection Center under the collection number CGMCC 1.16311. The original organism was isolated using a previously described method (7). In our study, V. mediterranei 117-T6 was pathogenic to the conchocelis of several Pyropia species, including P. yezoensis (Y. He, Q. Liu, M. Xu, Z. Tao, and R. Yang, unpublished data). Pyropia species are popular edible red algae with great commercial importance and have a long history of cultivation in northeast Asia. In this paper, the complete genome sequence of V. mediterranei strain 117-T6 was determined to facilitate further research into its virulence factors.

V. mediterranei 117-T6 was cultured in tryptic soy broth (TSB) medium containing 1% NaCl at 28°C for 12 h with shaking at 120 rpm. The genomic DNA (gDNA) was extracted using the Ezup column bacteria gDNA purification kit (Sangon Biotech Co., Ltd., Shanghai, China) according to the manufacturer’s protocols. The gene library was constructed using a SMRTbell template prep kit 1.0 and was quality controlled using Qubit 3.0 (Life Technologies, CA, USA) and a 2100 bioanalyzer (Agilent, Santa Clara, CA, USA). After library preparation, the genome was sequenced by single-molecule real-time sequencing (SMRT) using the PacBio sequel system (Pacific Biosciences, Menlo Park, CA) (8). The generated subreads were assembled using Hierarchical Genome Assembly Process (HGAP) version 4 with default parameters (9). The genome structure was annotated using Glimmer version 3.02 (10). To obtain the protein function annotation, the predicted protein sequences were compared with those in the nonredundant protein (NR), Cluster of Orthologous Groups (COG), KEGG, Gene Ontology (GO), and Swiss-Prot databases in NCBI using BLASTP with an E value of 1e−5 (11).

A total of 178,807 filtered subreads with a total length of 1,332,991,254 bp were generated after sequencing (average subread length, 7,454 bp; subread *N*_50_, 9,729 bp; 233-fold coverage). The reads were assembled, and we found that the complete genome sequence of V. mediterranei 117-T6 consists of two circular chromosomes (3,698,726 bp and 2,081,621 bp, respectively) and one plasmid (195,680 bp). The total length of V. mediterranei 117-T6 was 5,976,027 bp with a GC content of 44.04%, including 5,539 coding DNA sequences (CDS), 31 rRNAs, 115 tRNAs, and 264 noncoding RNAs (ncRNAs). The number of tandem repeat finder (TRF) repeats of chromosome1, chromosome2, and the plasmid were 48, 20, and 9, respectively, and the number of simple sequence repeats (SSRs) were 9, 3, and 2, respectively. The plasmid contained 2 clustered regularly interspaced short palindromic repeat (CRISPR) sequence cassettes.

### Data availability.

The complete genome sequence for V. mediterranei 117-T6 has been deposited in DDBJ/ENA/GenBank under the accession numbers CP033577 to CP033579 (BioProject number PRJNA498774). The raw data have been submitted to the Sequence Read Archive (SRA) under run number SRR8294759.
